# FEEDING PRACTICES IN THE FIRST YEAR OF LIFE: CHALLENGES TO FOOD AND NUTRITION POLICIES

**DOI:** 10.1590/1984-0462/2020/38/2018401

**Published:** 2020-07-29

**Authors:** Lorena dos Santos Tinôco, Clélia de Oliveira Lyra, Tamires Carneiro de Oliveira Mendes, Yan Nogueira Leite de Freitas, Adriana Souza da Silva, Ana Maria Silva Souza, Maria Ângela Fernandes Ferreira

**Affiliations:** a Centro Universitário do Rio Grande do Norte, Natal, RN, Brazil.; b Universidade Federal do Rio Grande do Norte, Natal, RN, Brazil.; c Universidade Federal do Amazonas, Manaus, AM, Brazil.

**Keywords:** Infant nutrition, Breast feeding, Feeding behavior, Public health, Nutrição do lactente, Aleitamento materno, Comportamento alimentar, Saúde pública

## Abstract

**Objective:**

To evaluate the feeding practices for infants under one year of age, according to food and nutrition policies.

**Methods:**

This is a descriptive cross-sectional study based on secondary data from the *Chamada Neonatal* project (research on prenatal, childbirth, and infant care) in the state of Rio Grande do Norte. The sample analyzed comprised 837 mother/child (under one year of age) pairs. We found a prevalence of data on exclusive breastfeeding (EBF) in the first hour of life - partial and total -, as well as on food consumed by children 24 hours prior to the interview. We estimated the probability of consumption according to the child’s age in days using the probit analysis.

**Results:**

Among the interviewed mothers, 64.8% (95%CI 62.4-70.8) declared breastfeeding in the first hour of life, and 60% (95%CI 56.41-63.07) of the children were still breastfed at the end of their first year of life. The median duration of EBF was 63 days (95%CI 60-67). Water or tea, dairy products, fruits, and vegetables were introduced early, with medians lower than 180 days. The probit analysis revealed that the consumption of breast milk tended to decrease and food intake to increase as the child gets older, with exponential growth in the “unhealthy food” group.

**Conclusions:**

Although most children were breastfed up to one year of life, few did so exclusively. Foods were introduced early, with increased consumption of unhealthy ones, resulting in inadequate dietary quality according to recommendations from food and nutrition public policies.

## RESUMO

**Objetivo:**

Avaliar as práticas alimentares em menores de um ano de idade, de acordo com as políticas de alimentação e nutrição.

**Métodos:**

Estudo transversal descritivo, com dados secundários da Chamada Neonatal no Estado do Rio Grande do Norte. A amostra analisada foi de 837 pares mãe/filho menor de um ano de idade, e observou-se a prevalência de dados do Aleitamento Materno Exclusivo (AME), na primeira hora de vida, parcial e total, assim como dos alimentos ingeridos pelas crianças nas últimas 24 horas anteriores à entrevista. Por meio da análise de probitos, estimaram-se as probabilidades de consumo dos alimentos por idade da criança, em dias.

**Resultados:**

A prática de aleitamento na primeira hora de vida foi relatada por 64,8% (IC95% 62,4-70,8) das mães entrevistadas e, ao final do primeiro ano de vida, 60% (IC95% 56,41-63,07) das crianças ainda estavam sendo amamentadas. A mediana de aleitamento exclusivo foi de 63 dias (IC95% 60-67). Água ou chá, alimentos lácteos, frutas, legumes e verduras foram introduzidos precocemente, com medianas menores que 180 dias. Observou-se por análise de probitos que o consumo de leite materno tendeu a diminuir e o de alimentos a aumentar, de acordo com a idade da criança, com aumento exponencial do grupo “alimentos não saudáveis”.

**Conclusões:**

Apesar de a maioria das crianças ser amamentada até um ano de vida, poucas estavam em aleitamento exclusivo. Alimentos foram introduzidos precocemente, com aumento do consumo dos não saudáveis, resultando em inadequação da qualidade alimentar frente ao preconizado pelas políticas públicas de alimentação e nutrição.

## INTRODUCTION

Children up to 6 months of age do not need teas, juices, other types of milk, or even water.^1^ The introduction of liquids or solids before six months decreases the duration and frequency of breastfeeding (BF), interferes with the absorption of important nutrients, such as iron, reduces the efficiency of lactation in the intergestational period, and increases infant mortality and morbidity.^2^

After the sixth month of age, the infant should be introduced to appropriate complementary feeding, but BF should continue until the second year of life or more. At six months, parents and/or guardians should offer food to the child, starting with those of pasty consistency (salty and fruit purees) until they can eat family meals, which should occur as of the eighth month.^3^

Since the 1980s, the Brazilian Ministry of Health (MoH) is concerned with the promotion of BF and the quality of complementary feeding for children younger than two years. The MoH, in collaboration with the Pan-American Health Organization (PAHO), established the “Ten steps to a healthy diet for Brazilian children under two years” to guide mothers and health professionals.^4^ Subsequently, the MoH, through the General Coordination of Food and Nutrition Policies (*Coordenação-Geral da Política de Alimentação e Nutrição* - CGPAN), proposed the National Strategy for Healthy Complementary Feeding (*Estratégia Nacional para Alimentação Complementar Saudável* - ENPACS) as an instrument to strengthen actions that support and promote healthy complementary feeding in the public health system (*Sistema Único de Saúde* - SUS).^3^

Despite all scientific evidence that proves the superiority of BF over other diets for infants under one year of age and the existence of food and nutrition public policies targeted at children, BF rates and the dietary quality in Brazil, particularly in the Northeast region, are well below the recommended standards, contributing to the high levels of infant mortality and morbidity still found in the country.^5^ Therefore, knowing the profile of food introduction is important to promote healthy eating habits, such as the continuity of BF.^6^ Thus, this study aimed to evaluate the feeding practices for infants under one year of age, according to recommendations from food and nutrition public policies.^3^ ^,^ ^4^ ^,^ ^7^ ^,^ ^8^ ^,^ ^9^

## METHOD

This cross-sectional study used the database of national population-based research entitled *Chamada Neonatal: avaliação da atenção ao pré-natal, ao parto e aos menores de um ano na Amazônia Legal e no Nordeste, Brasil, 2010*,^10^ which evaluated prenatal, childbirth, and infant care in the Legal Amazon and Northeastern Brazil, involving mothers and children younger than one year who participated in the first stage of a multi-vaccination campaign carried out in 252 municipalities signatories to the Pact for the Reduction in Infant Mortality, in June 12, 2010. In this study, we used data from nine municipalities of Rio Grande do Norte (Natal, Currais Novos, Mossoró, Caicó, Pau dos Ferros, São Gonçalo do Amarante, Macaíba, Ceará-Mirim, and Parnamirim), which had priority in the pact.^10^

The calculation of the sample size considered a prevalence of 35.9% for the outcome “any complication during pregnancy” in Rio Grande do Norte, since investigating BF was not the main goal of *Chamada Neonatal*. We assumed a 4.0% error and a 95% confidence interval (95%CI). The sample comprised 837 mother/child pairs (482 from the capital Natal and 355 from the inland), who were recruited using a two-stage cluster sampling: the first randomly selected the vaccination stations, and the second determined a fraction of the draw for each station for the systematic selection in the vaccination queue.^10^

This research included only children under one year of age, living in the same city as the vaccination station where the study was conducted, non-twins, and those who were not adopted. If the mother had two children under one year of age, the youngest was recruited for the study, in an attempt to reduce the mother’s recall bias.

The data collection instrument was a standard form prepared and pre-tested by the researchers of *Chamada Neonatal*, administered in the vaccination stations or home visits (in this case, only for children under three months of age not accompanied by the mother, who lived in the capitals).

The dependent variables were obtained based on the following indicators:

- BF in the first hour of life: we considered positive answers those reporting that the child was breastfed soon after birth, in the first hour of life.
- Exclusive BF (EBF): we considered positive answers those declaring the consumption of breast milk in the 24 hours prior to the interview and denying the intake of one of the other foods listed, as recommended by the World Health Organization (WHO). The limit of 24 hours was used to reduce the recall bias of the participant.
- Partial BF (PBF): when the child received breast milk and other types of milk in the 24 hours prior to the interview.
- BF: when the child consumed breast milk (directly from the breast or by milking), regardless of receiving other foods.

We also considered complementary feeding as a dependent variable, evaluated according to food groups with common nutritional characteristics, based on recommendations from the MoH,^4^ resulting in eight food groups: breast milk; water or tea; fruits and vegetables (fruit juice, fruit, vegetables, açaí berry); dairy products (porridge with milk, other types of milk); grains and tubers (porridge without milk, potato/cassava/yam/arracacha, cassava flour); refined grains (crackers/cookies/bread/cakes); “unhealthy foods” (packaged snacks, soft drinks, hard candies/bonbons/lollipops/candies); and family meals.

The independent variables were the child’s age, maternal age, maternal schooling, place of residence (capital or inland), adherence to a federal welfare program, and the child’s age, in days, at the introduction of food groups, estimated by the probit analysis.^11^

We tabulated the data in the software Stata 12.0, and a descriptive analysis enabled us to characterize the sample according to the variables investigated. In addition, we calculated the probability of food introduction based on the child’s age, in days, using the probit analysis.^12^

## RESULTS

In this study, 837 mother/child (under one year of age) pairs were interviewed. Table 1 presents the distribution of the sample according to the characteristics of the study population.

**Table 1:** Weighted distribution* of the study population in nine municipalities signatories to the Pact for the Reduction in Infant Mortality and participating in *Chamada Neonatal*, according to sociodemographic characteristics. Rio Grande do Norte, 2010.

Variables	Categories	n	%	95%CI
Child’s gender	Female	408	48.8	45.1-52.5
	Male	429	51.2	47.5-54.9
	Total	837	100	
Child’s age	>6 months	403	48.1	43.6-52.6
	<6 months	434	51.9	47.4-56.4
	Total	837	100	
Maternal age	<20 years	145	17.5	15.9-19.2
	20 to 29 years	458	55.2	52.5-57.9
	≥30 years	226	27.3	24.7-30.2
	Total	829	100	
Maternal schooling	Incomplete elementary school (0-7 years)	202	24.3	19.2-30.2
	Complete elementary school (8-10 years)	257	31.0	26.3-36.1
	Complete high school (≥11 years)	371	44.7	35.7-54.1
	Total	830	100	
Maternal ethnicity	White	279	33.4	29.7-37.4
	Multiracial	444	53.2	48.9-57.5
	Black	94	11.2	9.4-13.2
	Asian and indigenous	18	2.2	-4.6-9.0
	Total	835	100	
Head of the family	Mother	199	23.8	18.7-29.8
	Someone else	636	76.2	70.2-81.3
	Total	835	100	
Participant in any welfare program	No	591	70.9	67.2-74.6
	Yes	243	29.1	23.4-34.8
	Total	834	100	
Place of residence	Capital	482	57.6	53.2-62.0
	Inland	355	42.4	37.3-47.5
	Total	837	100	

95%CI: 95% confidence interval; *weighted to represent the proportional participation of each child in the total sample in each municipality evaluated, according to the 2010 census distribution.

Among the interviewed mothers, 64.8% (95%CI 62.4-70.8) declared breastfeeding in the first hour of life, with a higher percentage in the capital (66.6%; 95%CI 66.6-68.6). However, the percentage of EBF stands out (20%; 95%CI 14.1-25.8). In addition, at the time of the interview, PBF was practiced by over half of the mothers (55.1%; 95%CI 49.3-60.7), and BF by 75.9% (95%CI 73.0-78.8). We also underline that the median duration of EBF and BF was 63 days (95%CI 60-67) and 358 days (95%CI 353-364), respectively.

**Figure 1:**
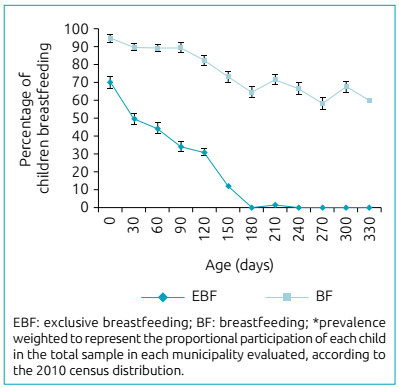
Percentage* of breastfeeding, exclusive and total, in nine municipalities signatories to the Pact for the Reduction in Infant Mortality and participating in *Chamada Neonatal*, according to the child’s age (days). Rio Grande do Norte, 2010.

Figure 1 shows the percentage of EBF and BF according to the child’s age in days. It demonstrates that almost all children were breastfed in the first 30 days of life, the reduction in the percentage of BF was slow and gradual, and, close to the end of the first year of life, 60% (95%CI 56.41-63.07) of the children were still breastfed. In contrast, EBF had a much smaller rate: in the first 30 days of life, over half of the children were not receiving breast milk exclusively, a percentage that increased with each passing day: 70% at 120 days, almost 90% at 150 days, and 100% at 180 days.

**Figure 2:**
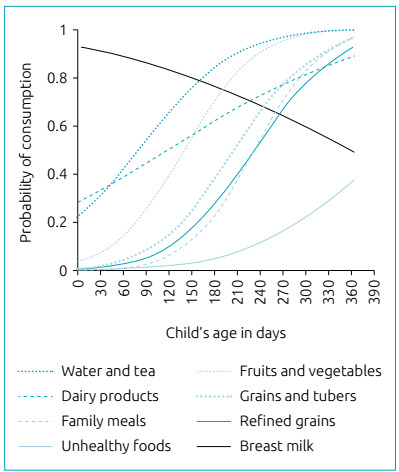
Probability of consumption by age, in days, in children younger than one year from nine municipalities signatories to the Pact for the Reduction in Infant Mortality and participating in *Chamada Neonatal*. Rio Grande do Norte, 2010.

Table 2 reports the median ages at the introduction of these food groups, in days, and Figure 2 depicts the probability curves of food introduction, according to the child’s age, in days, calculated by the probit analysis.

**Table 2:** Median age, in days, at the introduction of foods to children younger than one year from nine municipalities signatories to the Pact for the Reduction in Infant Mortality and participating in *Chamada Neonatal*. Rio Grande do Norte, 2010.

Food	Median (days)	95%CI
Water and tea	80	75.6-84.3
Fruits and vegetables	141	137.7-144.3
Dairy products	119	111.0-127.0
Grains and tubers	208	204.4-211.6
Refined grains	234	230.2-237.8
“Unhealthy foods”*	364	362.4-365.6
Family meals	234	230.9-237.1

95%CI: 95% confidence interval; *packaged snacks, soft drinks, hard candies/bonbons/lollipops/candies.

## DISCUSSION

This study, which has a considerable sample compared to other similar investigations,^13^ ^,^ ^14^ ^,^ ^15^ ^,^ ^16^ reveals some satisfactory BF practices in Rio Grande do Norte, with most mothers breastfeeding in the first hour of life and most children under one year of age still being breastfed. Nevertheless, actions, programs, and strategies to reduce the prevalence of early introduction of other foods, especially unhealthy ones, are necessary.

In 2009, 67.7% of children from Brazilian capitals and the Federal District were breastfed in the first hour of life,^17^ while in Natal, capital of Rio Grande do Norte, this percentage was 66.6% in 2009 and 2010, as stated by this research. Still, the percentage found in Natal is considered good, according to WHO.^10^ General data from Northeastern Brazil revealed that BF in the first hour of life, step four of the Baby-Friendly Hospital Initiative (BFHI), was a protective factor in the group of children under six months of age, providing immunological and probiotics components.^10^ ^,^ ^8^

The median duration of EBF shows that the children evaluated started the weaning process too early, since more than half of them was not exclusively breastfeeding in the first month of life, a median much lower than the one recommended by WHO.^1^ On the other hand, WHO classifies the percentage of EBF identified in this work as reasonable^7^. The prevalence of EBF in children younger than six months rarely exceeds 50%, as reported in more recent studies: 28.0% in Piauí,^12^ 4.0% in Minas Gerais,^15^ 34.8% in Rio de Janeiro,^16^ and in a systematic review by Uema et al.^13^

As to the duration of BF, over half of the children were breastfed until almost one year of life, even with the early introduction of other types of milk. WHO proposes that BF continues, even after the introduction of complementary feeding, until the second year of life.^1^ The duration of BF in Brazil has been increasing: 72 days in 1975, 165 days in 1989, 210 days in 1996,^7^ and 342 days in 2008.^17^ In Rio Grande do Norte, the duration of BF was 231 days in 1999,^18^ increasing to 330 days in 2008.^17^ In 2010, the state had 28 days added to the median BF, which remains above the national average.^17^

The median duration of EBF increased by approximately one month in Brazil, going from 23.4 days, in 1999,^18^ to 54.1 days, in 2008.^17^ In Rio Grande do Norte, specifically in Natal, this interval grew from 25 to 56 days (in 1999^18^ and 2008,^17^ respectively), with an increment of 10 days in 2010.^7^ These increases may be the result of public policies to promote BF, which enabled the government to enhance its educational BF campaigns, train professionals from Family Health Teams (*Equipes de Saúde da Família* - ESF), and, consequently, improve prenatal care in the primary health care system.^8^ Nevertheless, the results remain unsatisfactory: most of the recent studies show much smaller median EBF than the recommended by WHO.^13^

The consumption of water and teas tends to increase and remain constant as the child gets older. In 2011, Lopes and Polônio^19^ revealed that water was introduced to 95% of the children assessed and tea to 55%. Simon et al.^20^ identified a median of 28 days for the introduction of these liquids to children aged 6 to 12 months. This early introduction might be due to cultural aspects, as some mothers believe that these liquids are necessary when the child is thirsty and to prevent dehydration.^9^ In the Northeast, teas are used as “medicine” in the case of colic and gas.^21^

Fruits and vegetables constituted another food group introduced early. The probability of consumption of these foods increases with age but tends to remain constant in the last quarter before the 12^th^ month of age. In this study, the median introduction of this food group was around the fifth month, when almost 90% of infants were no longer exclusively breastfed. Data from the 2006/2007 National Demographic and Health Survey (*Pesquisa Nacional de Demografia e Saúde* - PNDS) revealed that the North and Northeast regions had the lowest percentages of fruit and vegetable consumption.^22^

Fruits and vegetables are ingredients of sweet and salty purees, which, according to the guideline from the MoH,^4^ should be offered to infants only after they reach six months of age. These foods can provide greater variety and bioavailability of vitamins and minerals for children, encouraging healthy eating habits, as recommended by the Global Strategy on Diet, Physical Activity and Health^23^. Thus, even knowing that most children older than six months consumed fruits and vegetables, it was not possible to ascertain if this intake is frequent and in sufficient quantities, as we only evaluated the consumption of these foods in the 24 hours prior to the interview.

The Brazilian Society of Pediatrics does not recommend cow milk for infants under one year of age because of its allergenic properties; high protein content; inadequate proportion between casein and whey proteins; high levels of sodium; and insufficient amounts of carbohydrates, essential fatty acids, vitamins, iron, and minerals for this age.^23^ In the population studied, the introduction of dairy products occurred before 180 days, with the possibility of this consumption increasing with age, while the intake of breast milk decreased in each quarter of life. In 2003, Simon et al.^20^ found that, as the child gets older, their likelihood of receiving other types of milk and porridge is greater than that of the receiving breast milk, with a median introduction of non-breast milk of 60 days. In addition to the ease of access to non-breast milk and thickeners among the most vulnerable populations, this choice might also be rooted in maternal culture.^23^

Between the 6^th^ and 12^th^ month of life, the child needs to adapt to the new foods, whose flavors, textures, and consistencies are very different from those of breast milk, including grains and tubers, which increase the energy density and provide protein.^4^ The median introduction of these foods was satisfactory according to recommendations from WHO^1^ and MoH,^4^ with the probability of consumption increasing with age, unlike the results from Simon et al.,^20^ who detected a median introduction of this food group before 180 days of life. Regional and seasonal foods should be offered to these infants, and the Northeast region has great availability of grains and tubers, such as cassava, yam, and potato.^24^

Among high-energy foods, we identified that a considerable percentage of children consumed cakes, cookies, bread, and crackers, the so-called “refined grains,” as they have white flour in their composition^25^ - a result also found in Bahia^21^ and Paraíba.^26^ Even when introduced after the sixth month, the probability of introduction of these foods increases with age, and, in the fourth quarter of life, the percentage of consumption of these products is higher than that of dairy products and breast milk among infants, favoring the accumulation of body fat.

This study revealed a median introduction of family meals, in days, a little lower than the recommended after the eighth month (240 days) of life.^4^ Step eight of the “Ten steps to a healthy diet for Brazilian children under two years” proposes to “avoid sugar, coffee, canned foods, fried foods, soft drinks, hard candies, snacks, and other candies in the first years of life, and use salt moderately.”^4^ This study showed that the intake of foods considered “unhealthy”^4^ had a median introduction of around 11 months. We also noted that the probability of consumption of these foods presents an exponential growth curve, that is, with a constant tendency to increase with age. The growing consumption of “unhealthy foods” is quite worrying, since they only provide excess calories and have little nutritional value, in addition to including substances not recommended for the age, such as sugars in large quantities, coloring agents, and preservatives present in ultra-processed foods. Thus, they increase the risk of obesity, currently considered an epidemic in Brazil, and dental caries.^6^ ^,^ ^26^ ^,^ ^27^ Moreover, the sodium found in these foods can stimulate children to even greater consumption of other foods rich in this substance, leading to them keeping this habit until adulthood, raising the risk of hypertension.^28^

The limitations of the study include its cross-sectional design, which did not allow us to assess causality between the feeding practices for children younger than one year, and the social vulnerability of the population. However, the knowledge acquired in this research enabled us to describe the baseline for future comparisons and/or discussions. Considering the concern with infant mortality, the feeding practices for children younger than one year may represent a reflection point, given that the quality of care provided to the mother-child dyad constitutes an important tool for the performance of actions to improve health indicators.^29^

We also underline that presenting and discussing data collected in 2010 on the feeding practices for infants younger than one year born in Northeastern Brazil is extremely relevant, since the last population-based study carried out in Brazil with this purpose was the II National Survey on the Prevalence of Breastfeeding, in 2008.^17^ Therefore, exploring the data from *Chamada Neonatal* is necessary, as they represent a safe possibility of obtaining relevant information about an extremely vulnerable population, which could become the basis for further studies of this nature.

Thus, although most children were breastfed up to one year of life, few younger than six months did so exclusively. We identified the early introduction of foods, with increased consumption of “unhealthy” ones, resulting in inadequate dietary quality according to recommendations from food and nutrition public policies. Faced with this reality, it is crucial to strengthen policies on popular health education in communities and the continuing education of primary health care professionals, who are essential to promoting BF and guiding families on healthy complementary feeding and the opportune moment for its introduction.
